# HPMA-Based Polymer Conjugates for Repurposed Drug Mebendazole and Other Imidazole-Based Therapeutics

**DOI:** 10.3390/polym13152530

**Published:** 2021-07-30

**Authors:** Martin Studenovský, Anna Rumlerová, Libor Kostka, Tomáš Etrych

**Affiliations:** Institute of Macromolecular Chemistry, Czech Academy of Sciences, Heyrovský Sq. 2, 162 06 Prague 6, Czech Republic; annarumlerova@centrum.cz (A.R.); kostka@imc.cas.cz (L.K.); etrych@imc.cas.cz (T.E.)

**Keywords:** mebendazole, drug delivery, drug repurposing, polymer, HPMA, controlled release

## Abstract

Recently, the antitumor potential of benzimidazole anthelmintics, such as mebendazole and its analogues, have been reported to have minimal side effects, in addition to their well-known anti-parasitic abilities. However, their administration is strongly limited owing to their extremely poor solubility, which highly depletes their overall bioavailability. This study describes the design, synthesis, and physico-chemical properties of polymer-mebendazole nanomedicines for drug repurposing in cancer therapy. The conjugation of mebendazole to water-soluble and biocompatible polymer carrier was carried out via biodegradable bond, relying on the hydrolytic action of lysosomal hydrolases for mebendazole release inside the tumor cells. Five low-molecular-weight mebendazole derivatives, differing in their inner structure, and two polymer conjugates differing in their linker structure, were synthesized. The overall synthetic strategy was designed to enable the modification and polymer conjugation of most benzimidazole-based anthelmintics, such as albendazole, fenbendazole or albendazole, besides the mebendazole. Furthermore, the described methodology may be suitable for conjugation of other biologically active compounds with a heterocyclic N-H group in their molecules.

## 1. Introduction

### 1.1. Benzimidazole Derivatives in Antitumor Therapy

The development of new antitumor drugs has been limited by the high cost of experimental and clinical trials required by the US Food and Administration (FDA) and other health institutions. Drug repurposing, i.e., investigation of existing and already approved drugs with known toxicity profile and pharmacokinetics for novel therapeutic indications [1] should therefore bypass the elevated economic burden of trials and speed up the drug approval process.

**Figure 1:**
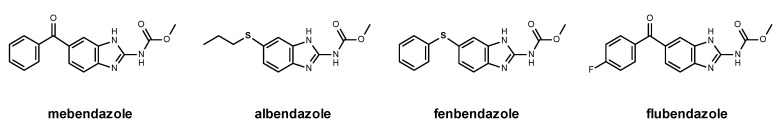
Chemical structures of benzimidazole derivatives.

Benzimidazoles have long been used as broad-spectrum anthelmintics. However, some benzimidazole derivatives exhibit antitumor potential [2,3,4,5]. Namely mebendazole (MBZ) and albendazole (ABZ), already in use in human medicine, and flubendazole (FLZ) and fenbendazole (FBZ), prescribed in veterinary practice, are the most promising anticancer drug candidates (Figure 1). They inhibit tubulin assembly and suppress microtubule formation by binding to active colchicine sites of the β-tubulin subunit leading to mitotic arrest and apoptosis [3,6]. Thus, they can inhibit cell proliferation, angiogenesis, metastatic activity, and show synergism with other chemotherapeutics (e.g., docetaxel) or radiotherapy [6,7,8]. Since their molecular size is quite small, they are not substrates for P-glycoprotein (P-gp) and multidrug resistance should not occur during their use. Moreover, MBZ has been described as a P-glycoprotein (P-gp) inhibitor [9], thus MBZ and its analogs appear to be promising antitumor chemotherapeutics. However, the persisting problem with benzimidazole derivatives is their extremely poor water solubility, which severely restricts their bioavailability and thus makes it difficult to reach serum concentrations required for antitumor activity.

### 1.2. Mebendazole Mechanism of Action and Antitumor Potential

Mebendazole is a benzimidazole derivative with extremely low water solubility and thus very poor bioavailability (~17–22% of oral dose reaches the blood circulation with significant inter-individual variation in pharmacokinetics) [10,11]. This is probably the reason why MBZ in per oral treatment does not exert any serious side effects. Clearance of MBZ from organism is mainly via metabolites in bile and feces, with only 2–5% excretion via urine [12].

Mebendazole has been used as an anthelmintic drug since 1974; however, its antitumor activity took almost 30 years to be published [6]. Ever since, various studies have described the cytostatic activity of MBZ in a wide range of tumor types e.g., breast, ovary, and colorectal carcinoma, glioblastoma, gastric cancer, melanoma or lung carcinoma [2,6,13,14,15]. Either as a single antitumor agent or in combination with other chemotherapeutics or radiotherapy, MBZ treatment inhibits tumor progression together with a decrease of metastatic spread and therefore improved survival. Moreover, it is worth mentioning that MBZ treatment also stimulates antitumor immune response [16,17].

The main mechanism of MBZ cytostatic activity is presumably its ability to interfere with microtubule apparatus of tumor cells resulting in defective cellular structures, intracellular transport, and glucose metabolism [18]. In vitro studies showed inhibition of factors involved in tumor progression upon MBZ treatment, e.g., decrease of matrix metalloproteinase-2 activity, inhibition of antiapoptotic activity of Bcl-2, inhibition of multidrug resistance protein transporters P-gp or MRP1, or inhibition of angiogenesis [9,13,19,20]. However, in vivo studies have not fully confirmed in vitro observations [21,22]. Several studies reported potential side effects of high dose MBZ treatment, including abdominal pain, diarrhea, and in rare cases neutropenia, marrow aplasia, alopecia, and elevation of transaminases levels [23,24].

### 1.3. Drug Delivery Systems Based on Synthetic Copolymers

Considering current knowledge, developing a general approach of effective cancer therapy would require the design of drugs with strong antitumor activity, concurrently with minimal adverse side effects. Importantly, polymer-based high-molecular-weight (HMW) delivery systems have been recently recognized as nanosystems with potent anticancer efficacies devoid of significant systemic toxicity [25,26]. Thus, the concept of HMW drug delivery systems, including water-soluble polymers such as *N*-(2-(hydroxypropyl)methacrylamide (HPMA) copolymers, has become generally accepted. Preferably, low-molecular-weight (LMW) drugs are covalently bound to a polymer via spacers, which enables the controlled release of the active drug in target tissues or cells. Polymer drug carriers are designed to alter the drug biodistribution and optimize its pharmacokinetics. Binding of the LMW drug to the HMW carrier results in its prolonged blood circulation, enhanced accumulation in solid tumors, controlled drug release, reduction of systemic toxicity and immunogenicity, and possibility in induction of resistance against tumor regrowth upon treatment [27,28]. Moreover, the binding to the polymer carrier can solubilize and stabilize water-insoluble drugs before biotransformation and degradation. The HMW anticancer drug delivery systems rely on passive accumulation in solid tumors based on the enhanced permeability and retention (EPR) effect, which is caused by the morphological and physiological differences between healthy and malignant tissues [29]. Numerous studies have shown higher permeability of tumor blood vessels compared to normal blood vessels [30]. The endothelial lining of the capillaries in the tumor is fenestrated and leaky, and thereby permeable to macromolecules. Moreover, tumor lymphatic drainage is mostly defective or even missing; therefore, macromolecules are retained within tumor tissues. The discovery of the EPR phenomenon [31] led to the development of tumor-selective delivery of polymer conjugates, micellar and liposomal drugs, and gene vectors. In case of HPMA copolymer-based drug conjugates, the EPR effect was observed after administration of conjugates with a molecular weight (*M*_w_) exceeding 20,000 g·mol^−1^ and increased with the higher *M*_w_ of the polymer [32].

### 1.4. Polymer-Drug Linkers Based on a Prodrug Strategy

**Figure 2:**
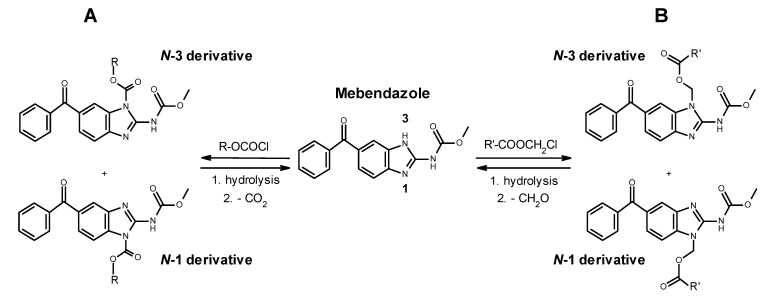
Generation and cleavage of carbamate (**A**) and acyloxymethyl (**B**) derivatives of mebendazole (MBZ). Due to a tautomerism of the MBZ molecule (1,3-H shift), a statistical mixture of *N*-1 and *N*-3 derivatives is formed in all cases. The presence of both isomers was indicated by doubled high-performance liquid chromatography (HPLC) peaks and nuclear magnetic resonance (NMR) signals of carbamate protons. For better clarity, only *N*-3 isomers will be depicted in the next figures.

The linkage between drugs and polymer carriers must be generally sufficiently stable in the bloodstream but capable of releasing the parent drug within the target site. While the structure of the polymer precursor can be tailor-made, the structure of the drug is well established, which determines the probability of chemical modification towards its attachment to the polymer. Thus, for example, active compounds containing hydroxyl can be bound via ester, ether, or acetal linker, while amines can be transformed to amide, urethane or carbamate, carboxyl to ester, and carbonyl to acetal or hydrazone. A great inspiration for a design of polymer-drug conjugates can be found in the prodrug chemistry [33], the broad field of pharmacochemistry. The prodrug strategy is in principle the same as for the polymer-drug conjugate: appropriate modification of the parent drug with the aim to enhance its pharmacokinetics as well as pharmacodymamics. In other words, the polymer-drug conjugates may be a high-molecular-weight prodrug. Nitrogen-containing heterocyclic compounds, like mebendazole and other benzimidazole anthelmintics, can be reversibly modified via the nitrogen atom [34]. In this study, carbamate and acyloxymethyl group, both cleavable by lysosomal hydrolases, were utilized for the preparation of LMW mebendazole derivatives as well as its polymer conjugates. The general formation and reverse degradation of the derivatives is shown in Figure 2.

## 2. Materials and Methods

### 2.1. Chemicals

Acetic acid, β-alanine, 1-aminopropan-2-ol, 3-azidopropanol, 2,2′-azobis(2-methylpropionitrile), 6-(Boc-amino)hexanoic acid, chloromethyl chlorosulfate, chloromethyl pivalate, dibenzocyclooctyne-amine, dicyclohexylcarbodiimide, 4-(dimethylamino)pyridine, ethyl chloroformate, ethyldiisopropylamine, 1-ethyl-3-(3-dimethylaminopropyl)carbodiimide hydrochloride, methacryloyl chloride, mebendazole, 4-nitrobenzyl chloroformate, phosgene solution, silica gel, sodium hydride, tetrabutylammonium hydrogen sulfate, tetramethylurea, thiazolidine-2-thione, and trifluoroacetic acid were purchased from Sigma-Aldrich (Prague, Czech Republic). Acetonitrile, methanol and other common solvents and chemicals were purchased from Merck (Prague, Czech Republic). All chemicals and solvents were of analytical grade.

### 2.2. Analytical Methods

Analyses were performed on a high-performance liquid chromatograph (HPLC; Shimadzu, Japan) using a reverse-phase column (Chromolith Performance RP-18e 100 × 4.6 mm) with UV detection. A mixture of water and acetonitrile was used as the eluent at a gradient 0–100 vol.% and a flow rate of 2 mL/min. Elemental composition was determined using a Perkin Elmer Elemental Analyzer 2400 CHN (Perkin Elmer, Waltham, MA, USA). Nuclear magnetic resonance (NMR) spectra were measured on a Bruker Avance MSL 300 MHz NMR spectrometer (Bruker Daltonik, Bremen, Germany). Molecular weights *M*_w_ and *M*_n_ of the polymers were determined by gel permeation chromatography (GPC) using an HPLC Shimadzu system equipped with a GPC column (TSKgel G3000SWxl 300 × 7.8 mm; 5 μm), UV–Vis, refractive index (RI) Optilab^®^-rEX and multiangle light scattering (MALS) DAWN EOS (Wyatt Technology Co., Goleta, CA, USA) detectors using a methanol:sodium acetate buffer (0.3 M; pH 6.5) mixture (80:20 vol.%; flow rate 0.5 mL/min). Preparative HPLC chromatography was performed on PrepChrom C-700 (Büchi, Flawil, Switzerland). UV–Vis spectra were measured on Specord 205 (Analytik Jena AG, Jena, Germany). Mass spectra (MS) were measured on an ion trap mass spectrometer LCQ Fleet (Thermo Scientific, Waltham, MA, USA). The hydrodynamic diameter (*D_h_*) of the polymer conjugates in PBS buffer pH 7.4 (5 mg/mL, 25 °C) was measured using a Nano-ZS instrument (ZEN3600, Malvern, UK). The intensity of scattered light was detected at angle θ = 173° using a laser with a wavelength of 632.8 nm. Data were evaluated using the DTS (Nano) program. The values were the mean of at least five independent measurements.

### 2.3. Syntheses

#### Mebendazole Derivatives **1**–**5**

**Figure 3:**
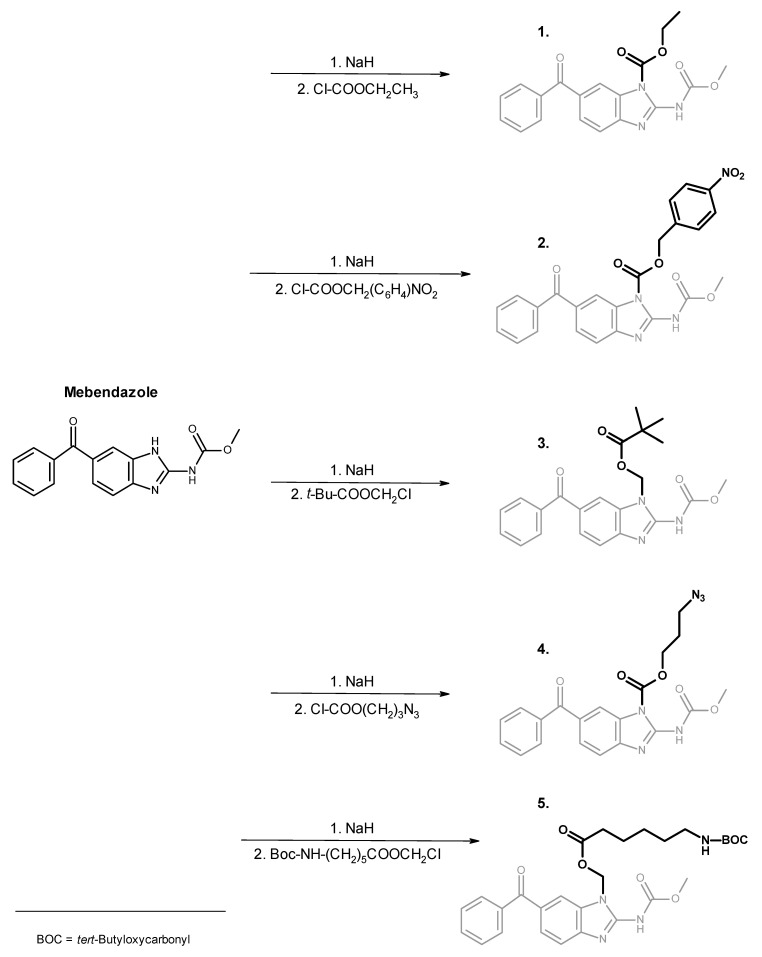
Synthesis of model mebendazole derivatives **1**–**3** and reactive derivatives **4**, **5** designed for the attachment to a polymer carrier.

A general synthetic procedure for low-molecular-weight of mebendazole is described in Figure 3.

### 2.4. N-Ethoxycarbonyl Mebendazole (**1**)

Mebendazole (100 mg, 0.34 mmol) was suspended in 3 mL of tetramethylurea and ethyl chloroformate (200 mg, 1.9 mmol) was added. The reaction mixture was stirred until homogenous and then the solvent was removed under vacuum. The residuum was dissolved in dichloromethane and the traces of unreacted mebendazole were removed by triple extraction with 5% HCl. The organic layer was dried under anhydrous magnesium sulfate, filtered, and the solvent was removed under vacuum. The crude product was crystallized from ethyl acetate and dried under vacuum. Yield: 43 mg (34%), 98% pure (HPLC, 220 nm). MS: The corresponding molecular peak M/Z = 390 (M + Na) was detected. Elemental analysis: calcd. C 62.12, H 4.66, N 11.44%, found C 62.05, H 4.7, N 11.32%. ^1^H NMR 300 MHz (DMSO-*d*_6_, 297 K): 1.26 t (3H, CH_3_CH_2_), 3.63 s (3H, OCH_3_), 4.26 q (2H, CH_3_CH_2_), 7.56–7.84 m (8H, Ar), 12.38 + 12.61 s (1H, NH).

### 2.5. N-(4-Nitrobenzyloxycarbonyl) Mebendazole (**2**)

Mebendazole (100 mg, 0.34 mmol) was suspended in 4 mL of tetramethylurea and 4-nitrobenzyl chloroformate (150 mg, 0.7 mmol) was added. The reaction mixture was stirred until homogenous and then the solvent was removed under vacuum. The residuum was dissolved in dichloromethane, filtered, and any remaining traces of mebendazole were removed by triple extraction with 5% HCl. The organic layer was dried under anhydrous magnesium sulfate, filtered, and the solvent was removed under vacuum. The crude product was crystallized from a mixture of hexane/dichloromethane 1:1 and dried under vacuum. Yield: 34 mg (21%), 97% pure (HPLC, 220 nm). MS: The corresponding molecular peak M/Z = 497 (M + Na) was detected. Elemental analysis: calcd. C 60.76, H 3.82, N 11.81%, found C 60.04, H 3.88, N 11.72%. ^1^H NMR 300 MHz (DMSO-*d*_6_, 297 K): 3.63 s (3H, OCH_3_), 5.18 s (2H, ArCH_2_), 7.51–8.17 m (12H, Ar), 12.35 + 12.59 s (1H, NH).

### 2.6. N-Pivaloyloxymethyl Mebendazole (**3**)

Mebendazole (1 g, 3.4 mmol) and sodium hydride (200 mg of 60% oil suspension, 5 mmol) were suspended in 20 mL of dimethylformamide and sonicated until a clear yellow solution of mebendazole sodium salt was formed. Chloromethyl pivalate (700 mg, 4.7 mmol) was added and the reaction mixture was stirred overnight. The solvent was removed under vacuum, the residuum was dissolved in dichloromethane, filtered, concentrated under vacuum, and separated on the silica gel in ethylacetate/dichloromethane/hexane 5:2:5 with 1% of trifluoroacetic acid. Yield: 378 mg (27%), 99% pure (HPLC, 220 nm). MS: The corresponding molecular peak M/Z = 432 (M + Na) was detected. Elemental analysis: calcd. C 64.54, H 5.66, N 10.26%, found C 64.24, H 5.75, N 10.31%. ^1^H NMR 300 MHz (DMSO-*d*_6_, 297 K): 1.1 s (9H, *t*-Bu), 3.65 s (3H, OCH_3_), 6.08 s (2H, NCH_2_), 7.55–7.85 m (8H, Ar), 12.34 + 12.5 s (1H, NH).

### 2.7. N-(3-Azidopropyloxycarbonyl) Mebendazole (**4**)

3-Azidopropanol (211 mg, 2.09 mmol) and sodium carbonate (1 g, 9.4 mmol) were mixed in 3 mL of dichloromethane and 20% phosgene solution in toluene (5 g, 10.1 mmol) was added. The reaction mixture was stirred for 2 h, filtered and the solvents were evaporated under vacuum. The obtained 3-azidopropyl chloroformate was added without further purification to a solution of sodium salt of mebendazole (617 mg mebendazole equiv., 2.09 mmol) in dimethyl formamide (see above) and the solution was stirred for 2 h. The reaction was then quenched with 0.5 mL of trifluoroacetic acid, and the volatiles were evaporated under vacuum. The residuum was dissolved in dichloromethane, filtered and the remaining unreacted mebendazole was removed by triple extraction with 5% HCl. The organic layer was dried under anhydrous magnesium sulfate, filtered, and the solvent was removed under vacuum. The crude product was then purified on silica gel in a mixture of dichloromethane and acetone 20:1. Yield: 240 mg (29%), 97.5% pure (HPLC, 220 nm). MS: The corresponding molecular peak M/Z = 445 (M + Na) was detected. Elemental analysis: calcd. C 56.87, H 4.3, N 19.9%, found C 57.05, H 4.37, N 20.2%. ^1^H NMR 300 MHz (DMSO-*d*_6_, 297 K): 1.84 qu (2H, CH_2_CH_2_N_3_), 3.31 t (2H, CH_2_N_3_) 3.63 s (3H, OCH_3_), 4.15 t (2H, OCH_2_), 7.54–7.84 m (8H, Ar), 12.41 + 12.6 s (1H, NH).

### 2.8. N-(6-(Boc-amino)hexanoyloxymethyl) Mebendazole (**5**)

6-(Boc-amino)hexanoic acid (604 mg, 2.6 mmol), chloromethyl chlorosulfate (350 mg, 2.12 mmol), sodium hydrogen carbonate (870 mg, 10.36 mmol) and tetrabutylammonium hydrogen sulfate (90 mg, 0.27 mmol) were vigorously stirred in a 20 mL of dichoromethane/water emulsion for 24 h. The organic layer was separated, extracted five times with brine, and dried with anhydrous sodium sulfate. The sodium sulfate was filtered off and the solvent was removed from the filtrate under vacuum. The crude chloromethyl 6-(Boc-amino)hexanoate was then added without further purification to a solution of sodium salt of mebendazole (755 mg mebendazole equiv., 2.56 mmol) in dimethyl formamide (see above), and stirred for 24 h. The reaction mixture was concentrated under vacuum, filtered, and separated on a C-18 reversed phase preparative chromatogram in a water/acetonitrile gradient. Yield: 440 mg (32%), 98.5% pure (HPLC, 220 nm). MS: The corresponding molecular peak M/Z = 561 (M + Na) was detected. Elemental analysis: calcd. C 62.44, H 6.36, N 10.4%, found C 62.15, H 6.37, N 10.25%. ^1^H NMR 300 MHz (DMSO-*d*_6_, 297 K): 1.19 qu (2H, CH_2_CH_2_CH_2_N), 1.31 qu (2H, CH_2_CH_2_CO), 1.34 s (9H. *t*-Bu), 1.47 qu (2H, CH_2_CH_2_N), 2.32 t (2H, CH_2_CO), 2.83 t (2H, CH_2_N), 3.65 s (3H, OCH_3_), 6.08 s (2H, NCH_2_-O), 6.72 t (1H, NHCOOt-Bu), 7.54–7.82 m (8H, Ar), 12.32 + 12.51 s (1H, NHCOOMe).

### 2.9. Polymer Conjugates of Derivatives **4** (Conjugate **I**) and **5** (Conjugate **II**)

**Figure 4:**
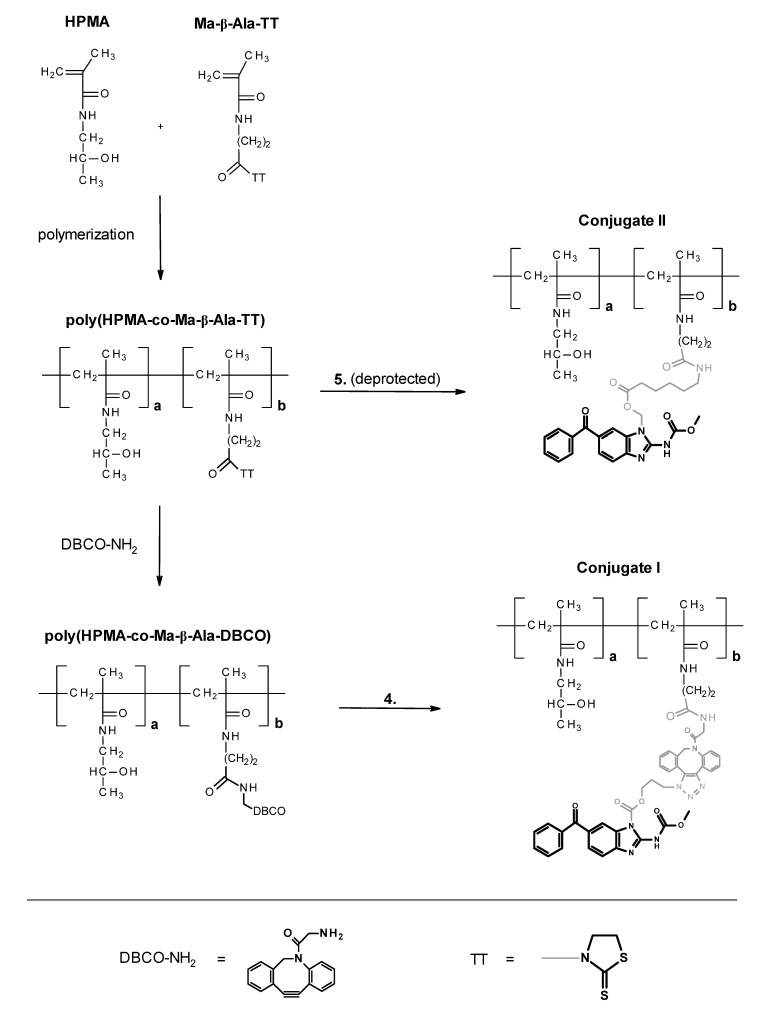
Synthesis of HPMA-based mebendazole polymer conjugates **I** (carbamate linker) and **II** (ester linker).

A general synthetic procedure for polymer conjugates of mebendazole is described in Figure 4.

### 2.10. N-(2-Hydroxypropyl)methacrylamide (HPMA)

Synthesis of this monomer was carried out according to literature [35] via reaction of methacryloyl chloride with 1-amino propan-2-ol. Yield: 75%, 99% pure (HPLC, 220 nm). Elemental analysis: calcd. C 58.72, H 9.15, N 9.78%, found C 58.90, H 9.10, N 9.88%.

### 2.11. 3-(3-Methacrylamidopropanoyl)thiazolidine-2-thione (Ma-β-Ala-TT)

Synthesis of this reactive monomer was carried out according to literature [36] using a two-step procedure starting with condensation of methacryloyl chloride with β-alanine with consequent activation to aminoreactive thiazolidine-2-thione amide. Yield: 72%, 99% pure (HPLC, 220 nm). Elemental analysis: calcd. C 59.62, H 7.88, N 9.91%, found C 58.99, H 8.1, N 9.78%.

### 2.12. poly(HPMA-co-Ma-β-Ala-TT)

This reactive polymer precursor was prepared by controlled radical RAFT copolymerization (reversible addition-fragmentation chain-transfer polymerization) of HPMA and Ma-β-Ala-TT following the procedure described previously [36]. A molar ratio of monomers:CTA:initiator 400:2:1 was used. The molar ratio of HPMA:Ma-β-Ala-TT in the reaction mixture was 92:8. Characteristics: *M*_w_ = 35 kDa, *M*_n_ = 32 kDa, content of TT groups was determined spectrophotometrically in methanol (ε_305_ = 10,800 Lmol^−1^cm^−1^): 0.35 mmol/g.

### 2.13. poly(HPMA-co-Ma-β-Ala-DBCO)

poly(HPMA-*co*-Ma-β-Ala-TT) (1 g, 0.35 mmol of TT groups) was dissolved in 5 mL of dimethyl formamide and dibenzocyclooctyne-amine (100 mg, 0.38 mmol) was added. The reaction mixture was stirred for 2 h and the modified polymer was then precipitated into diethyl ether, dissolved in small amount of methanol, precipitated again into diethyl ether, filtered off, and dried under vacuum. Yield: 0.95 g, molecular weight (GPC): *M*_w_ = 40 kDa, *M*_n_ = 34 kDa, content of DBCO groups was determined spectrophotometrically in methanol (ε_292_ = 13,000 Lmol^−1^cm^−1^): 0.32 mmol/g.

### 2.14. Conjugate **I** (Polymer Bound Azido Derivative **4**)

poly(HPMA-*co*-Ma-β-Ala-DBCO) (0.5 g, 0.16 mmol of DBCO groups) was dissolved in 3 mL of dimethyl formamide and *N*-(3-azidopropyloxycarbonyl) mebendazole (**4**) (84.5 mg, 0.2 mmol) was added. The reaction mixture was stirred for 12 h and the polymer conjugate was then precipitated into diethyl ether, dissolved in methanol, precipitated again into diethyl ether, filtered off, and dried under vacuum. Yield: 0.44 g, molecular weight (GPC): *M*_w_ = 48 kDa, *M*_n_ = 41 kDa, the content of mebendazole was determined by HPLC after treatment of the conjugate with 1% NaOH solution for 30 min: 0.31 mmol/g (8.9 wt.%).

### 2.15. Conjugate **II** (Polymer Bound Amino Derivative **5**)

*N*-(6-(Boc-amino)hexanoyloxymethyl) mebendazole (**5**) (950 mg, 1.76 mmol) was dissolved in 28 mL of trifluoroacetic acid and the reaction mixture was shaken for 1 h. After complete deprotection (confirmed by HPLC) the acid was evaporated and the solid was co-distilled twice with 20 mL of acetonitrile. The resulted free *N*-(6-aminohexanoyloxymethyl) mebendazole was dissolved in 28 mL of dimethyl formamide and combined with a solution of poly(HPMA-*co*-Ma-β-Ala-TT) (7.4 g, 2.59 mmol TT) and ethyldiisopropylamine (1 g, 7.7 mmol). After 1 h of stirring, 1-aminopropan-2-ol (350 mg, 4.66 mmol) was added and the mixture was stirred for 15 min to quench the remaining polymer-bound TT groups. The conjugate was then precipitated into diethyl ether, filtered off, re-precipitated twice the same way and dried under vacuum. Yield: 7.8 g, molecular weight (GPC): *M*_w_ = 37 kDa, *M*_n_ = 33 kDa, content of mebendazole was determined by HPLC after treatment of the conjugate with 1% NaOH solution for 30 min: 0.22 mmol/g (6.45 wt.%).

### 2.16. Hydrolytic Stability of the Conjugates

A long-term circulation hydrolytic stability under blood-mimicking conditions was evaluated by incubation of the samples (1 mg/mL) in the PBS buffer in pH 7.4 at 37 °C for three days. The concentration of the released free mebendazole was determined by HPLC at 300 nm.

## 3. Results and Discussion

**Figure 5:**
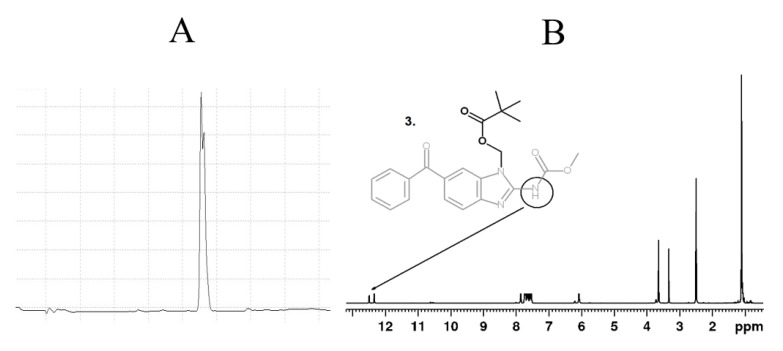
HPLC chromatogram at 300 nm (**A**) and ^1^H NMR spectrum of derivative **3** (**B**).

In this paper, a chemical modification of an anthelmintics mebendazole, a repurposed anticancer drug for parenteral administration, was designed and optimized. We aimed to synthesize polymer conjugates, which will take the benefit from the covalent attachment of mebendazole, and the linkage will be tailored for the tumor-cell specific degradation by the lysosomal hydrolases. We assumed that a such concept will bring a significant increase in the efficacy of the mebendazole, which will be specifically accumulated in the solid tumor via the EPR effect-base polymer accumulation and subsequently released to fully act as an anticancer drug. To reach that goal, three low-molecular-weight derivatives, carbamates **1**, **2** and pivaloyloxymethyl derivative **3**, were prepared to develop and optimize the multistep synthetic procedure prior to synthesis of corresponding polymer conjugates. The heterocyclic nitrogen atom of mebendazole was acylated or alkylated either after previous transformation to a sodium salt with sodium hydride or by an alternative route using tetramethylurea as a base/solvent [37]. The overall low to moderate yields, not exceeding 30%, were mainly due to a need of chromatographic separation. The same synthetic strategy was used for derivatives **4** and **5**, already designed for direct conjugation to a polymer. Compound **4** contained an enzymatic cleavable carbamate group between the mebendazole moiety and the spacer azide-terminated for attaching to the polymer precursor via copper-free click chemistry. Compound **5** comprised a cleavable ester group between the drug and the spacer, which is terminated via protected amine for subsequent aminolytic attachment to a polymer precursor bearing TT groups. As illustrated in Figure 2, all mebendazole derivatives were mixtures of *N-1* and *N-3* isomers due to a 1,3-H shift in the mebendazole molecule. There were no attempts to separate the individual isomers because of their almost identical properties in view of chromatographic separation and chemical reactivity. Figure 5 shows a HPLC chromatogram and ^1^H NMR spectrum of derivative **3**, where the doubled peak and N–H singlet, respectively, indicate a presence of both isomers.

**Figure 6:**
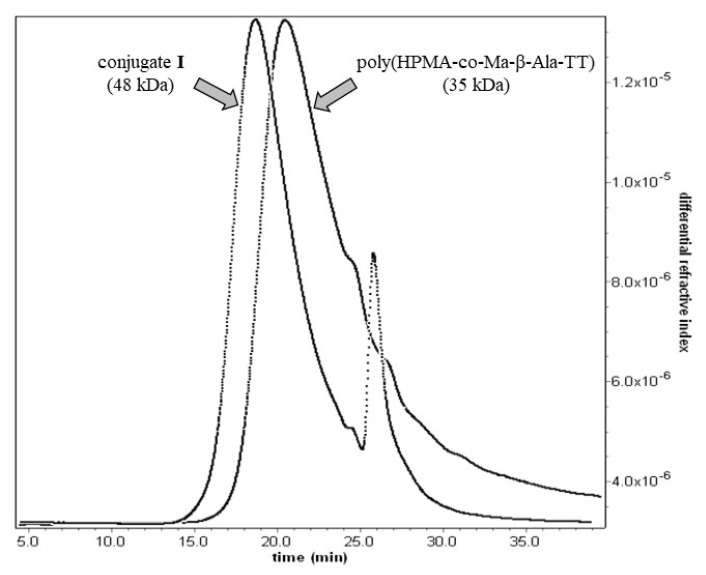
Comparison of GPC curves of poly(HPMA-*co*-Ma-β-Ala-TT) and conjugate **I**.

**Figure 7:**
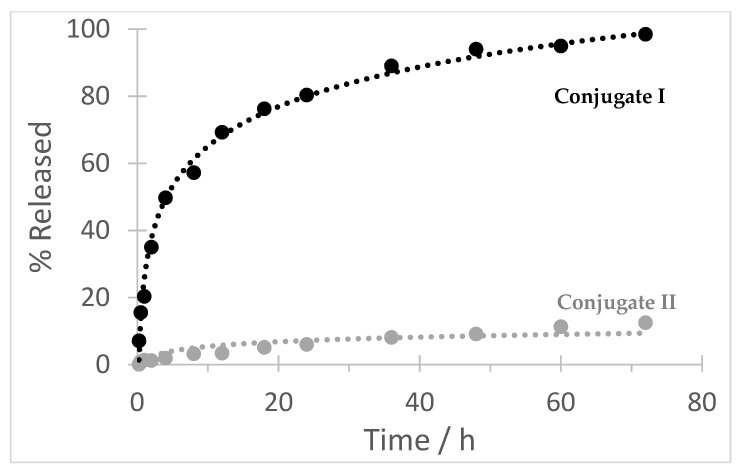
Stability of conjugates **I** and **II** in a buffer pH 7.4.

Importantly, controlled radical RAFT polymerization [38,39] was employed for the preparation of reactive polymer precursors. This approach enables to obtain polymers with precisely set molecular weight, with narrow distribution of molecular weight and thus minimize the fraction exceeding the renal threshold (~50 kDa). poly(HPMA-*co*-Ma-β-Ala-TT) was synthesized by copolymerization of HPMA and Ma-β-Ala-TT co-monomers where the content of TT groups and the average molecular weight were set by concentration of the monomers and the initiator in a reaction mixture. The typical values are 0.35 mmol TT groups per gram and *M*_w_ ~35 kDa (Figure 1). To obtain the conjugate **I**, the poly(HPMA-*co*-Ma-β-Ala-TT) was first modified with DBCO-amine to introduce the reactive DBCO groups. The introduction of DBCO groups did not change the physico-chemical properties of the polymer poly(HPMA-*co*-Ma-β-Ala-DBCO) when compared to its polymer precursor. In the next step, the derivative **4** was clicked in a copper free manner to obtain conjugate **I.** Here, *M*_w_ increases to 48 kDa (Figure 6), with the increase of the hydrodynamic size to 16.3 nm. We assume such an increase of the hydrodynamic size of polymer conjugate **I.** to the concurrent presence of the hydrophobic DBCO groups and mebendazole, which led to the formation of the nanoaggregates with hydrophobic core composed of DBCO and mebendazole and hydrophilic shell formed by the water-soluble copolymer. Such behavior would be beneficial in a forthcoming biological evaluation as the increased size of the conjugate **I.** would lead to an enhanced tumor accumulation. Moreover, after the delivery and release of the mebendazole polymer carrier, of which the size will be dropped back to the value close to the limit of renal threshold should be easily removed from the organism via urine excretion. On the contrary, conjugate **II** was prepared by direct aminolytic reaction of poly(HPMA-*co*-Ma-β-Ala-TT) with freshly deprotected mebendazole derivative **5** with subsequent quenching of remaining TT groups with 1-aminopropan-2-ol. Here, no significant increase in both the molecular weight and hydrodynamic size was observed. The conjugate **II** remained water-soluble and no sign of aggregation was observed. The mebendazole content in all conjugates ranged between 6 wt.% and 9 wt.% and was determined by HPLC after alkaline hydrolysis. This procedure was optimized to be effective and quantitative for the precise mebendazole determination without any undesirable side reactions. A treatment in 1% NaOH water solution satisfied the above-mentioned criteria. Characterization of all prepared polymers is summarized in Table 1. The key feature of all the drug delivery systems is based on the sufficient stability during the blood circulation to the site of the action. Any premature release of a carried drug led to the lowering of the drug delivery efficacy, which can cause significant increase in the side effects on healthy organs. Thus, the stability of the conjugates was comprehensively investigated under blood stream mimicking conditions. Figure 7 shows a time dependent release of free mebendazole in PBS buffer in pH 7.4 at 37 °C from conjugates **I** and **II**, mimicking their stability during blood circulation. The data clearly show that the carbamate bond in conjugate **I** is cleaved much faster at blood pH (t_50%_ ~4 h) in contrast to the ester bond in conjugate **II** (t_10%_ ~53 h). In other words, the ester linker is more stable under blood-mimicking slightly alkaline conditions and would be therefore more suitable for evaluation in vivo. Importantly, even the formation of nanoaggregates in the case of conjugate I., where the localization of mebendazole in the inner hydrophobic core is expected, did not cause significant reduction of the hydrolytic cleavage of the carbamate bond. Based on these results, the chemical and biological properties of polymer conjugate **II** will be further evaluated in a future study.

It is worth noting that the synthetic strategy described in the study can be applied for conjugation in a wide range of other therapies to a water-soluble or amphiphilic biocompatible polymers to expand the family of macromolecular drugs in a broad therapeutic portfolio. In addition to benzimidazole anthelmintics, any active compound with a heterocyclic N-H group in the molecule, can be involved. For example, pyrimidine or purine antimetabolites with a well-established antineoplastic activity (5-Fluorouracil, 6-Mercaptopurine, 6-Thioguanine), indole derivatives (anticancer agents Ellipticine, Vincristine or Panobinostat, antimigrenic drug Sumatriptan, anti-HIV drug Ateviridine) or other compounds, such as antineoplastics Vemurafenib and Axitinib, may be of interest.

## 4. Conclusions

In summary, two chemically distinct linkers, carbamate and ester, for attachment of the repurposed drug mebendazole to the HPMA-based polymer carrier, were designed and comprehensively studied. Five low-molecular-weight MBZ derivatives were designed and their step-by-step synthesis was optimized. On that basis, carbamate and ester-based polymer conjugates were prepared and their solution behavior and stability in blood-mimicking environment was evaluated. The ester linkage between the MBZ and polymer carrier is, in contrast to the carbamate one, long-term stable and has all the attributes to be selected for attachment of all benzimidazole-based anthelmintics. Further, a combination of hydrophobic and bulky DBCO units with mebendazole residues causes a significant increase in hydrodynamic diameter, which can be beneficial in view of a more pronounced EPR effect. Moreover, we envision that the proposed synthetic strategy may be employed in other biologically active nitrogen heterocycles containing drugs to water soluble polymer carriers. From a clinical point of view, the results of this work allow for use of the above-mentioned anticancer potential of mebendazole by its solubilization, improving the pharmacokinetics and controlled release. The strategy developed can be also utilized for a broad spectrum of other drugs which further increases the clinical relevance of this study.

## Figures and Tables

**Table 1:** Properties of polymer precursors and polymer conjugates.

Sample	*M*_w_ kDa	*M*_w_/*M*_n_	Size (*D_h_*) nm	Functionality mmolg^−1^	Group Type
poly(HPMA-*co*-Ma-β-Ala-TT)	35	1.1	8.6	0.35	TT
poly(HPMA-*co*-Ma-β-Ala-DBCO)	40	1.2	10.5	0.32	DBCO
Conjugate **I**	48	1.2	16.3	0.31	MBZ
Conjugate **II**	37	1.1	10.3	0.22	MBZ

## Data Availability

The data presented in this study are available on request from the corresponding author.

## References

[1] Cha Y., Erez T., Reynolds I.J., Kumar D., Ross J., Koytiger G., Kusko R., Zeskind B., Risso S., Kagan E. (2018). Drug repurposing from the perspective of pharmaceutical companies. Br. J. Pharmacol..

[2] Mukhopadhyay T., Sasaki J., Ramesh R., Roth J.A. (2002). Mebendazole elicits a potent antitumor effect on human cancer cell lines both in vitro and in vivo. Clin. Cancer Res..

[3] Chu S.W., Badar S., Morris D.L., Pourgholami M.H. (2009). Potent inhibition of tubulin polymerisation and proliferation of paclitaxel-resistant 1A9PTX22 human ovarian cancer cells by albendazole. Anticancer Res..

[4] Dogra N., Kumar A., Mukhopadhyay T. (2018). Fenbendazole acts as a moderate microtubule destabilizing agent and causes cancer cell death by modulating multiple cellular pathways. Sci. Rep..

[5] Hou Z.J., Luo X., Zhang W., Peng F., Cui B., Wu S.J., Zheng F.M., Xu J., Xu L.Z., Long Z.J. (2015). Flubendazole, FDA-approved anthelmintic, targets breast cancer stem-like cells. Oncotarget.

[6] Sasaki J., Ramesh R., Chada S., Gomyo Y., Roth J.A., Mukhopadhyay T. (2002). The anthelmintic drug mebendazole induces mitotic arrest and apoptosis by depolymerizing tubulin in non-small cell lung cancer cells. Mol. Cancer Ther..

[7] Rushworth L.K., Hewit K., Munnings-Tomes S., Somani S., James D., Shanks E., Dufes C., Straube A., Patel R., Leung H.Y. (2020). Repurposing screen identifies mebendazole as a clinical candidate to synergise with docetaxel for prostate cancer treatment. Br. J. Cancer.

[8] Poruchynsky M.S., Komlodi-Pasztor E., Trostel S., Wilkerson J., Regairaz M., Pommier Y., Zhang X., Maity T.K., Robey R., Burotto M. (2015). Microtubule-targeting agents augment the toxicity of DNA-damaging agents by disrupting intracellular trafficking of DNA repair proteins. Proc. Natl. Acad. Sci. USA.

[9] Celestino Pinto L., Moreira-Nunes C.D.F.A., Moreira Soares B., Rodrigues Burbano R.M., Rodrigues de Lemos J.A., Carvalho Montenegro R. (2017). Mebendazole, an antiparasitic drug, inhibits drug transporters expression in preclinical model of gastric peritoneal carcinomatosis. Toxicol. In Vitro.

[10] Dawson M., Braithwaite P.A., Roberts M.S., Watson T.R. (1985). The pharmacokinetics and bioavailability of a tracer dose of [3H]-mebendazole in man. Br. J. Clin. Pharmacol..

[11] Dawson M., Allan R.J., Watson T.R. (1982). The pharmacokinetics and bioavailability of mebendazole in man: A pilot study using [3H]-mebendazole. Br. J. Clin. Pharmacol..

[12] Dayan A.D. (2003). Albendazole, mebendazole and praziquantel. Review of non-clinical toxicity and pharmacokinetics. Acta Trop..

[13] Pinto L.C., Moreira Soares B., Viana Pinheiro J.J., Riggins G.J., Pimentel Assumpcao P., Rodriguez Burbano R.M., Carvalho Montenegro R. (2015). The anthelmintic drug mebendazole inhibits growth, migration and invasion in gastric cancer cell model. Toxicol. In Vitro.

[14] Bai R.Y., Staedke V., Aprhys C.M., Gallia G.L., Riggins G.J. (2011). Antiparasitic mebendazole shows survival benefit in 2 preclinical models of glioblastoma multiforme. Neuro Oncol..

[15] Simbulan-Rosenthal C.M., Dakshanamurthy S., Gaur A., Chen Y.S., Fang H.B., Abdussamad M., Zhou H., Zapas J., Calvert V., Petricoin E.F. (2017). The repurposed anthelmintic mebendazole in combination with trametinib suppresses refractory NRAS^Q61K^ melanoma. Oncotarget.

[16] Blom K., Senkowsky W., Jarvius M., Berglund M., Rubin J., Lenhammar L., Parrow V., Andersson C., Loskog A., Fryknas M. (2017). The anticancer effect of mebendazole may be due to M1 monocyte/macrophage activation via ERK1/2 and TLR8-dependent inflammasome activation. Immunopharmacol. Immunotoxicol..

[17] Blom K., Rubin J., Berglund M., Jarvius M., Lenhammar L., Parrow V., Andersson C., Loskog A., Fryknas M., Nygren P. (2019). Mebendazole-induced M1 polarisation of THP-1 macrophages may involve DYRK1B inhibition. BMC Res. Notes.

[18] Jornet D., Bosca F., Andreu J.M., Domingo L.R., Tormos R., Miranda M.A. (2016). Analysis of mebendazole binding to its target biomolecule by laser flash photolysis. J. Photochem. Photobiol. B.

[19] Doudican N., Rodriguez A., Osman I., Orlow S.J. (2008). Mebendazole induces apoptosis via Bcl-2 inactivation in chemoresistant melanoma cells. Mol. Cancer Res..

[20] Sung S.J., Kim H.K., Hong Y.K., Joe Y.A. (2019). Autophagy is a potential target for enhancing the anti-angiogenic effect of mebendazole in endothelial cells. Biomol. Ther..

[21] Williamson T., Bai R.Y., Staedtke V., Huso D., Riggins G.J. (2016). Mebendazole and a non-steroidal anti-inflammatory combine to reduce tumor initiation in a colon cancer preclinical model. Oncotarget.

[22] Zhang F., Li Y., Zhang H., Huang E., Gao L., Luo W., Wei Q., Fan J., Song D., Liao J. (2017). Anthelmintic mebendazole enhances cisplatin’s effect on suppressing cell proliferation and promotes differentiation of head and neck squamous cell carcinoma (HNSCC). Oncotarget.

[23] Fernández-Bañares F., Gonzalez-Huix F., Xiol X., Catala I., Miro J., Lopez N., Casais L. (1986). Marrow aplasia during high dose mebendazole treatment. Am. J. Trop. Med. Hyg..

[24] Colle I., Naegels S., Hoorens A., Hautekeete M. (1999). Granulomatous hepatitis due to mebendazole. J. Clin. Gastroenterol..

[25] Duncan R. (2009). Development of HPMA copolymer–anticancer conjugates: Clinical experience and lessons learnt. Adv. Drug Deliv. Rev..

[26] Kopeček J. (2013). Polymer-drug conjugates: Origins, progress to date and future directions. Adv. Drug Deliv. Rev..

[27] Rihova B., Kovar M. (2010). Immunogenicity and immunomodulatory properties of HPMA-based polymers. Adv. Drug Deliv. Rev..

[28] Sirova M., Kabesova M., Kovar L., Etrych T., Strohalm J., Ulbrich K., Rihova B. (2013). HPMA copolymer-bound doxorubicin induces immunogenic tumor cell death. Curr. Med. Chem..

[29] Maeda H. (2010). Tumor-selective delivery of macromolecular drugs via the EPR effect: Background and future prospects. Bioconjug. Chem..

[30] Taurin S., Nehoff H., Greish K. (2012). Anticancer nanomedicine and tumor vascular permeability; Where is the missing link?. J. Control. Release.

[31] Matsumura Y., Maeda H. (1986). A new concept for macromolecular therapeutics in cancer chemotherapy: Mechanism of tumoritropic accumulation of proteins and the antitumor agent smancs. Cancer Res..

[32] Seymour L.W., Miyamoto Y., Maeda H., Brereton M., Strohalm J., Ulbrich K., Duncan R. (1995). Influence of molecular weight on passive tumour accumulation of a soluble macromolecular drug carrier. Eur. J. Cancer.

[33] Simplício A.L., Clancy J.M., Gilmer J.F. (2008). Prodrugs for amines. Molecules.

[34] Zimmermann S.C., Tichy T., Vavra J., Dash R.P., Slusher C.E., Gadiano A.J., Wu Y., Jancarik A., Tenora L., Monincova L. (2018). N-substituted prodrugs of mebendazole provide improved aqueous solubility and oral bioavailability in mice and dogs. J. Med. Chem..

[35] Studenovsky M., Pola R., Pechar M., Etrych T., Ulbrich K., Kovar L., Kabesova M., Rihova B. (2012). Polymer carriers for anticancer drugs targeted to EGF receptor. Macromol. Biosci..

[36] Pola R., Janouskova O., Etrych T. (2016). The pH-dependent and enzymatic release of cytarabine from hydrophilic polymer conjugates. Physiol. Res..

[37] Luttringhaus A., Dirksen H.W. (1964). Tetramethylurea as a solvent and reagent. Angew. Chem. Int. Ed..

[38] Graeme M., Rizzardo E., Thang S.H. (2008). Radical addition–fragmentation chemistry in polymer synthesis. Polymer.

[39] Kostka L., Subr V., Laga R., Chytil P., Ulbrich K., Seymour L.W., Etrych T. (2015). Nanotherapeutics shielded with a pH responsive polymeric layer. Physiol. Res..

